# A comparative real-world analysis of finerenone and steroidal mineralocorticoid receptor antagonists in patients with chronic kidney disease

**DOI:** 10.3389/fphar.2026.1918516

**Published:** 2026-09-09

**Authors:** Daniele Vetrano, Giuseppe Cianciolo, Simona Barbuto, Danilo Ribichini, Sabrina Berti, Anna Mistretta, Martina Ginesi, Phillip Timothy Millar, Amedeo Grimaldi, Gisella Vischini, Elisabetta Poluzzi, Uberto Pagotto, Gaetano La Manna, Irene Capelli

**Affiliations:** 1 Department of Medical and Surgical Sciences (DIMEC), Alma Mater Studiorum University of Bologna, Bologna, Italy; 2 Nephrology, Dialysis and Kidney Transplant Unit, IRCCS Azienda Ospedaliero-Universitaria di Bologna, Bologna, Italy; 3 Division of Endocrinology and Diabetes Prevention and Care, IRCCS Azienda Ospedaliero-Universitaria di Bologna, Bologna, Italy; 4 UOC Nefrologia e Dialisi, Ospedale Santa Rosa (Belcolle), Viterbo, Italy; 5 Department of Neuroscience and Rehabilitation, University of Ferrara, Ferrara, Italy; 6 Nephrology and Dialysis Unit, Azienda Ospedaliero-Universitaria di Ferrara, Ferrara, Italy

**Keywords:** albuminuria, chronic kidney disease, drug safety, finerenone, heart failure, hyperkalemia, mineralocorticoid receptor antagonist, type 2 diabetes mellitus

## Abstract

**Background:**

Finerenone and steroidal mineralocorticoid receptor antagonists (sMRAs) reduce proteinuria in chronic kidney disease (CKD), but direct comparative data from routine clinical practice remain limited. The objective of this study was to compare the effectiveness, hemodynamic effects, kidney function trajectory, and safety of finerenone versus sMRAs in patients with proteinuric CKD in routine clinical practice.

**Methods:**

This retrospective single-center cohort study included adults receiving finerenone or an sMRA between January 2021 and May 2025. Longitudinal analyses included 174 patients with at least one post-baseline assessment (finerenone, n = 96; sMRA, n = 78). Changes in proteinuria, estimated glomerular filtration rate (eGFR), blood pressure, potassium, and glycated hemoglobin were assessed using mixed-effects models. A 1:1 propensity score-matched sensitivity analysis was performed.

**Results:**

Both treatments were associated with significant proteinuria reductions at 12 months, with percentage changes of −45.63% (95% confidence interval [CI], −59.42 to −27.15) with finerenone and −44.44% (95% CI, −60.08 to −22.66) with sMRAs, without a significant between-group difference (p = 0.927). Proteinuria reduction was already significant at 1 month with sMRAs, whereas it became significant from month 6 with finerenone. Both groups showed an early eGFR decline. At month 12, the decline remained significant in the sMRA group (−4.66 mL/min/1.73 m^2^; 95% CI, −7.35 to −1.97) but not in the finerenone group; however, no significant between-group difference in eGFR trajectory was observed. sMRAs produced a greater reduction in systolic blood pressure at 12 months than finerenone, with a between-group difference of 7.24 mmHg (95% CI, 2.25–17.66). Serum potassium increased in both groups without a significant between-group difference. Treatment discontinuation was less frequent with finerenone than with sMRAs (13.8% vs. 28.7%; p = 0.021). Findings for proteinuria and eGFR were consistent in the matched sensitivity analysis.

**Conclusion:**

In this real-world cohort, finerenone and sMRAs showed comparable antiproteinuric effectiveness. sMRAs were associated with a more pronounced blood pressure reduction, whereas finerenone showed more favorable renal profile, with lower treatment discontinuation and side effects.

## 1 Introduction

Chronic kidney disease (CKD) remains a major driver of cardiovascular morbidity, kidney failure, and premature death, particularly in patients with persistent albuminuria or overt proteinuria despite optimized renin–angiotensin-aldosterone system blockade (Jankowski et al., 2021; Claudel and Verma, 2025). Although renin-angiotensin-aldosterone system (RAAS) blockers, namely, angiotensin converting enzyme inhibitors (ACEi) and angiotensin receptor blockers (ARBs) have long represented the cornerstone of nephroprotective therapy, and sodium–glucose cotransporter 2 (SGLT2) inhibitors have further improved renal protection in recent years, substantial residual risk persists, and additional strategies to further reduce proteinuria and slow CKD progression are still needed (Giugliano et al.; Capelli et al., 2023). Mineralocorticoid receptor (MR) overactivation has emerged as a key pathophysiological pathway in CKD progression. In addition to its well-known role in sodium retention and blood pressure regulation, MR signalling in renal and cardiovascular cells beyond the classical aldosterone-sensitive epithelium, including podocytes, endothelial cells, fibroblasts, and inflammatory cells, contributes to kidney injury through oxidative stress, inflammation, vascular dysfunction, and fibrosis (Epstein et al., 2022). This broader view of MR biology provides a strong rationale for the use of MR antagonists (MRAs) in proteinuric kidney disease (Barrera-Chimal et al., 2022).

Steroidal MRAs (sMRA), such as spironolactone and eplerenone, have been used for years as add-on therapy in patients with resistant hypertension and proteinuric CKD, and prior studies and meta-analyses have shown that they can reduce blood pressure and albuminuria/proteinuria (Hu et al., 2023; Currie et al., 2016). However, their use in nephrology has been limited by concerns regarding hyperkalemia, endocrine adverse effects, and a more pronounced hemodynamic impact; accordingly, despite their long-standing off-label use in proteinuric CKD, sMRAs have not gained a formal place in major CKD guidelines as kidney-protective therapy (Richard et al., 2025).

Finerenone, a selective non-steroidal MRA (nsMRA), has expanded the therapeutic landscape by demonstrating cardiorenal benefit in patients with CKD and type 2 diabetes on top of optimized renin–angiotensin system (RAAS) inhibition (Zhai et al., 2024). In FIDELIO-DKD and FIGARO-DKD, and subsequently in the pooled FIDELITY analysis, finerenone reduced clinically relevant kidney and cardiovascular outcomes across a broad spectrum of diabetic CKD (Bakris et al., 2020; Pitt et al., 2021; Agarwal et al., 2022). Moreover, its cardiorenal benefits appear to be only marginally explained by blood pressure lowering, suggesting a therapeutic profile that is not merely hemodynamic (Ruilope et al., 2022). On this basis, current Kidney Disease: Improving Global Outcomes (KDIGO) guidance supports the use of a nsMRA in appropriately selected adults with CKD and type 2 diabetes, particularly in the presence of persistent albuminuria despite standard-of-care therapy (Kidney Disease: Improving Global Outcomes KDIGO CKD Work Group, 2024). More recently, the phase three FIND-CKD trial extended the evidence base for finerenone to non-diabetic CKD, showing a slower decline in eGFR than placebo over 32 months in adults without diabetes and with albuminuric CKD (Heerspink et al., 2026). In addition, the pooled individual participant data analysis INFINITY, integrating FIDELIO-DKD, FIGARO-DKD, and FIND-CKD across 14,574 participants, showed consistent kidney and cardiovascular benefits of finerenone across a broad spectrum of CKD, including reductions in hard clinical outcomes such as kidney failure, cardiovascular death, and all-cause mortality (Neuen et al., 2026).

At the same time, sMRAs have historically been used in selected patients with proteinuric CKD, particularly before finerenone became available or in settings where access to finerenone remains limited (Sarafidis et al., 2023). Despite this, direct comparative real-world data between steroidal and nonsteroidal MRAs remain scarce.

In this context, the objective of this study was to compare finerenone and sMRAs in a real-world cohort of patients with proteinuric CKD, with and without type 2 diabetes mellitus, and to characterize their longitudinal effects on proteinuria control, kidney function, blood pressure, potassium balance, and treatment tolerability over 12 months of routine nephrology care.

## 2 Materials and methods

### 2.1 Study design and setting

A retrospective, observational cohort study was conducted at IRCCS Azienda Ospedaliero-Universitaria Policlinico Sant’Orsola, Bologna, Italy, between January 2021 and May 2025. The study was carried out jointly by the Nephrology, Dialysis and Kidney Transplant Unit and the Diabetes Prevention and Care Unit.

The study was conducted in accordance with the principles of the Declaration of Helsinki and approved by the local Ethics Committee (REDIA study, ID 1008–2021, approved 15 December 2021).

### 2.2 Study population

Adult patients initiating either finerenone or an sMRA during the study period and with no prior exposure to any MRA were eligible for inclusion; patients undergoing therapeutic switching between steroidal and nonsteroidal MRAs were excluded.

The index date was defined as the date of MRA initiation, and patients were classified according to the treatment initiated at that date. For longitudinal analyses, only patients with at least one available post-baseline follow-up assessment were included. Baseline clinical and laboratory measurements were defined as the closest available assessments obtained within the 30 days preceding MRA initiation. Follow-up assessments were collected according to routine clinical practice and harmonized at approximately 1, 6, and 12 months after treatment initiation.

### 2.3 Data collection

Clinical, laboratory and treatment-related data were extracted from institutional electronic medical records. Baseline variables included demographic characteristics, comorbidities, blood pressure, body weight, renal function, proteinuria, potassium, glucose metabolism parameters, lipid profile, hemoglobin, and concomitant therapies, including renin-angiotensin-aldosterone system inhibitors (RAASi), SGLT2 inhibitors, glucagon-like peptide-1 (GLP-1) receptor agonists, and other glucose-lowering treatments where applicable.

Longitudinal data were collected at approximately 1, 6, and 12 months after treatment initiation, and longitudinal analyses were intentionally restricted to the first 12 months of follow-up to ensure a consistent observation period across treatment groups. For the sMRA cohort, additional subgroup analyses were performed according to the specific agent used, including eplerenone and canrenone.

## 3 Outcomes

The primary efficacy outcomes were longitudinal changes in proteinuria and estimated glomerular filtration rate (eGFR). Secondary analyses evaluated changes over time in hemodynamic, electrolyte, and metabolic parameters, including blood pressure, serum potassium, glucose, and glycated hemoglobin (HbA1c).

Additional correlation analyses assessed the relationship between the acute eGFR dip and short-term proteinuria reduction from baseline to month 1, total proteinuria reduction from baseline to the last available follow-up within the 12-month observation period, potassium change from baseline to month 1, and subsequent eGFR change from month 1 to month 12, separately within each treatment group. The acute eGFR dip was defined as the absolute decrease in eGFR from baseline to month 1, calculated as baseline eGFR minus month one eGFR and expressed in mL/min/1.73 m^2^, such that positive values represented a decline in kidney function.

Exploratory responder analyses were additionally performed. Proteinuria response was defined as a reduction of at least 30% or 50% from baseline, while systolic blood pressure (SBP) response was defined as a reduction of at least 10 mmHg from baseline. Composite good response renal response analyses, defined as a ≥30.0% reduction in proteinuria together with an eGFR decline at 12 months not exceeding 4.0 mL/min/1.73 m^2^, was also explored.

Safety analyses included treatment discontinuation and adverse events occurring during follow-up. Hyperkalemia categories were defined according to observed serum potassium values during follow-up as mild (>5.0 and <5.5 mmol/L), moderate (5.5–6.0 mmol/L), and severe (>6.0 mmol/L). Hypotension was defined as SBP <120 mmHg, and severe hypotension as SBP <100 mmHg. Renal safety was also explored through a ≥30% decline in eGFR from baseline, including analyses of early decline at 30 days and persistent decline when confirmed at a subsequent available follow-up.

Additional sensitivity analyses were prespecified to test the robustness of the main findings, including a 1:1 propensity score–matched analysis to reduce baseline treatment-selection imbalances and a descriptive analysis stratified by individual MRA type (eplerenone, canrenone, and finerenone) to explore the consistency of the observed patterns across specific treatment subgroups.

### 3.1 Statistical analysis

Continuous variables were summarized as median and interquartile range; categorical variables were reported as counts and percentages. Between-group comparisons at baseline were performed using parametric or non-parametric tests for continuous variables and chi-square test or Fisher’s exact test for categorical variables, as appropriate.

Longitudinal changes were analysed using mixed-effects models including treatment group, time, and treatment-by-time interaction, with patient-level random effects to account for repeated measures. For proteinuria, analyses were performed using log-transformed values, and results were back-transformed where appropriate for clinical interpretation. For each timepoint, model-based estimates, standard errors, comparisons versus baseline, and between-group differences with 95% confidence intervals were obtained. Overall effects of time, treatment group, and treatment-by-time interaction were also assessed.

Associations between the acute eGFR dip from baseline to month one and the prespecified changes in proteinuria, potassium, and subsequent eGFR were assessed separately within each treatment group using Spearman’s rank correlation analysis and graphically explored with scatter plots and fitted regression lines. Adjusted partial Spearman correlation analyses were additionally performed as sensitivity analyses, accounting for baseline eGFR, log-transformed baseline proteinuria, baseline RAAS inhibitor and SGLT2 inhibitor use, and, in the sMRA group, MRA molecule and baseline dose intensity. sMRA baseline dose intensity was defined within each individual agent rather than by absolute milligram dose, given the lack of direct dose equivalence between different steroidal MRAs. Eplerenone was categorized as low intensity at 25 mg/day and higher intensity at ≥50 mg/day, whereas canrenone was categorized as low intensity at 25 mg/day and higher intensity at ≥50 mg/day. The single patient treated with spironolactone was excluded from these adjusted analyses. Finerenone dose was not included because of the minimal between-patient variability in starting dose (10 mg for the majority of the cohort).

As a sensitivity analysis, patients treated with finerenone and sMRAs were matched 1:1 by propensity score using nearest-neighbor matching without replacement, according to age, sex, baseline eGFR, log-transformed baseline proteinuria, diabetes status, and baseline SGLT2 inhibitor use. After matching, the longitudinal trajectories of proteinuria and eGFR were re-evaluated in the matched sample using the same mixed-effects models applied in the primary analysis.

Statistical analyses were performed using R 4.5.3 (R Foundation for Statistical Computing, Vienna, Austria). A two-sided *p* value < 0.05 was considered statistically significant.

## 4 Results

### 4.1 Baseline characteristics

A total of 203 patients were identified (121 finerenone and 82 sMRA). Of those, 174 with available follow-up (96 finerenone and 78 sMRA) were included in the final analysis.

Baseline characteristics are summarized in Table 1. Overall, the cohort had a median age of 70.0 years and was predominantly male (75.3%). It was also characterized by a high prevalence of hypertension (96.9%), diabetes (81.0%), and cardiovascular disease (86.2%). Compared with the sMRA group, patients treated with finerenone were older (72.0 vs. 69.0 years, *p* = 0.02) and more frequently had diabetes (92.7% vs. 66.7%, *p* = 0.01). By contrast, the sMRA group had longer CKD duration (9.0 vs. 7.0 years, *p* = 0.005), higher BMI (30.9 vs. 28.7 kg/m^2^, *p* = 0.03), higher baseline proteinuria (2500.0 vs. 1500.0 mg/24 h, *p* = 0.001), and higher LDL-C levels (78.6 vs. 65.8 mg/dL, *p* = 0.03). Most other baseline clinical and biochemical variables were broadly comparable between groups.

**TABLE 1:** Baseline characteristics of the study population according to treatment group.

Variable	Overall N = 174	Steroidal MRA N = 78	Finerenone N = 96	P value
Age, y	70.0 [63.0–75.0]	69.0 [61.0–73.0]	72.0 [65.8–77.0]	0.02
Male sex	131/174 (75.3%)	55/78 (70.5%)	76/96 (79.2%)	0.2
Diabetes	141/174 (81.0%)	52/78 (66.7%)	89/96 (92.7%)	0.01
CVD	138/160 (86.2%)	57/70 (81.4%)	81/90 (90.0%)	0.1
Hypertension	159/164 (96.9%)	68/72 (94.4%)	91/92 (98.9%)	0.1
CKD duration, y	8.0 [5.0–11.0]	9.0 [6.0–13.0]	7.0 [4.0–9.5]	0.005
SBP, mmHg	130.0 [120.0–140.8]	135.0 [120.0–145.0]	130.0 [122.5–140.0]	0.8
DBP, mmHg	80.0 [70.0–80.0]	80.0 [70.0–80.0]	80.0 [70.0–80.0]	0.6
MAP, mmHg	96.7 [90.0–100.2]	96.7 [90.0–101.0]	96.7 [88.3–100.0]	0.8
Weight, kg	84.0 [72.0–96.0]	87.5 [76.0–100.5]	79.7 [69.2–95.1]	0.06
BMI, kg/m^2^	29.6 [25.8–32.9]	30.9 [26.9–33.6]	28.7 [25.4–31.3]	0.03
Creatinine, mg/dL	1.5 [1.2–1.9]	1.4 [1.1–2.0]	1.6 [1.2–1.9]	0.3
eGFR, mL/min/1.73 m^2^	45.9 [34.0–61.0]	50.0 [34.0–68.9]	43.0 [34.0–57.8]	0.2
Proteinuria, mg/24h	1899.0 [853.8–3131.2]	2500.0 [1193.0–4290.0]	1500.0 [702.5–2590.5]	0.001
Potassium, mmol/L	4.6 [4.2–4.8]	4.5 [4.2–4.8]	4.6 [4.3–4.9]	0.3
Glucose*, mg/dL	119.5 [95.8–140.0]	118.0 [93.0–137.2]	125.0 [103.5–140.2]	0.2
HbA1c*, mmol/mol	53.0 [48.0–61.2]	52.0 [47.5–59.0]	53.0 [48.0–63.0]	0.3
LDL-C, mg/dL	67.4 [52.8–95.1]	78.6 [55.2–114.0]	65.8 [48.3–83.2]	0.03
Hemoglobin, g/dL	13.6 [12.4–14.9]	13.1 [12.2–14.5]	13.8 [12.7–15.5]	0.01
RAASi	152/174 (87.4%)	65/78 (83.3%)	87/96 (90.6%)	0.1
SGLT2i	112/173 (64.7%)	46/77 (59.7%)	66/96 (68.8%)	0.2
GLP-1RA*	75/171 (43.1%)	33/78 (42.3%)	42/96 (43.8%)	0.8
DPP4-i*	25/167 (15.0%)	5/75 (6.7%)	20/92 (21.7%)	0.007
Finerenone	96/174 (55.1%)	-	96/96 (100%)	​
Canrenone	31/174 (17.8%)	31/78 (39.7%)	-	​
Eplerenone	46/174 (26.4%)	46/78 (59.0%)	-	​
Spironolactone	1/174 (0.6%)	1/78 (1.3%)	-	​

Values are expressed as median [interquartile range] or n/N (%), as appropriate. P values refer to comparisons between the steroidal MRA, and finerenone groups. Denominators may differ across variables because of missing data. Abbreviations: BMI, body mass index; CKD, chronic kidney disease; CVD, cardiovascular disease; DBP, diastolic blood pressure; DPP4-i, dipeptidyl peptidase-4, inhibitor; eGFR, estimated glomerular filtration rate; GLP-1RA, glucagon-like peptide-1, receptor agonist; HbA1c, glycated hemoglobin; LDL-C, low-density lipoprotein cholesterol; MAP, mean arterial pressure; MRA, mineralocorticoid receptor antagonist; RAASi, renin–angiotensin–aldosterone system inhibitor; SBP, systolic blood pressure; SGLT2i, sodium–glucose cotransporter two inhibitor. *available only in patients with Type 2 Diabetes.

### 4.2 MRA dosing and concurrent nephroprotective medications

**FIGURE 1:**
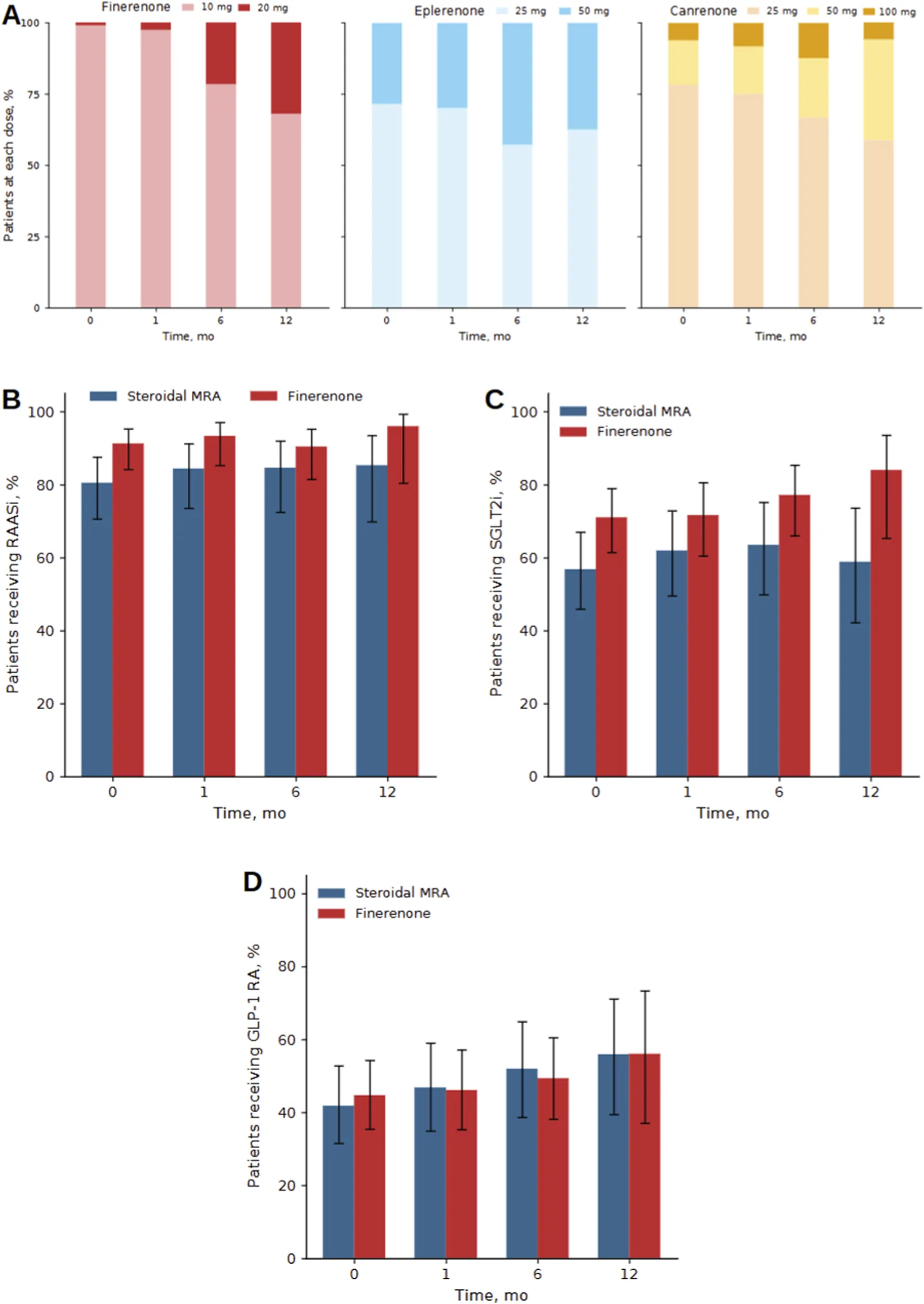
Longitudinal treatment exposure and concomitant nephroprotective therapy during follow-up. **(A)** Time-course distribution of MRA doses. **(B)** Longitudinal use of RAAS inhibitors, **(C)** SGLT2 inhibitors and **(D)** GLP-1 receptor agonists.

Across follow-up, the cohort was treated predominantly with low-dose MRA regimens (Figure 1A). In the finerenone group, 10 mg was used in nearly all patients at baseline and month one and remained the most frequent dose at months 6 and 12, although dose escalation to 20 mg became more common over time. Similarly, among sMRAs, eplerenone was prescribed mainly at 25 mg throughout follow-up, while canrenone was also used predominantly at 25 mg, with a gradual shift toward 50 mg at later time points; 100 mg was used only in a minority of patients at all visits. Overall, these findings indicate that most patients were managed with low-dose MRA therapy over time.

Concomitant nephroprotective therapies were commonly used in both groups. RAAS inhibitors were prescribed in the majority of patients throughout the study, with consistently high rates in both groups and numerically higher use in the finerenone group, particularly at baseline (90.6% vs. 83.3%) (Figure 1B). SGLT2 inhibitors were also frequently used, with a progressive tendency toward higher use in the finerenone group over time (84.0% vs. 58.8%) (Figure 1C). By contrast, GLP-1 receptor agonist use was broadly comparable between groups at all time points, remaining overall stable at around 40%–56% during follow-up (Figure 1D).

### 4.3 Longitudinal changes in clinical and laboratory variables

**FIGURE 2:**
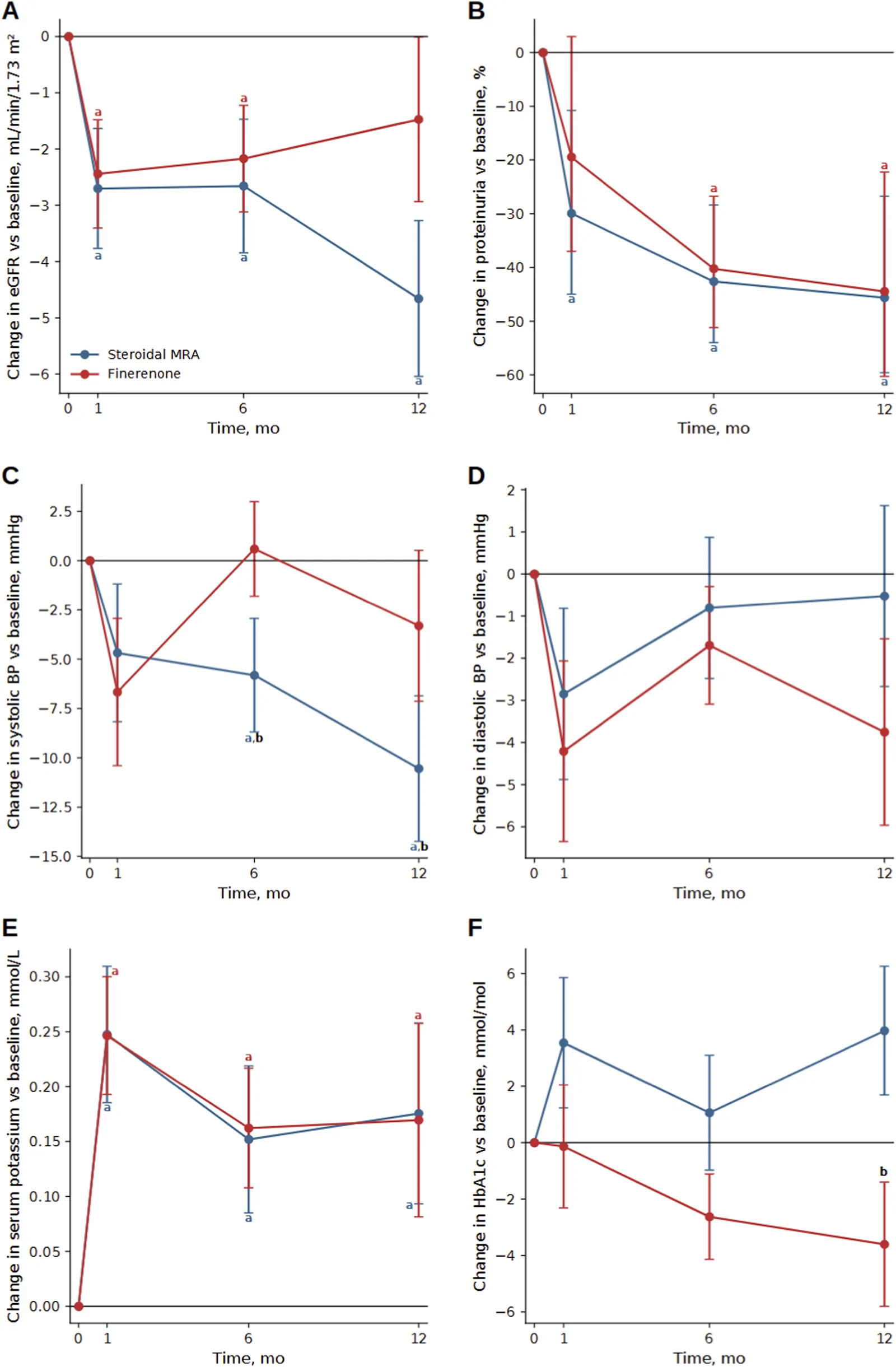
Longitudinal changes in kidney, hemodynamic, electrolyte, and metabolic parameters according to treatment group. **(A)** Change in eGFR, **(B)** percentage change in proteinuria, **(C)** change in SBP, **(D)** change in diastolic blood pressure, **(E)** change in serum potassium, and **(F)** change in HbA1c versus baseline over follow-up. Caption: Data are expressed as mean ± SE. a indicates *p* < 0.05 versus baseline at that time point; b indicates *p* < 0.05 for the between-group difference at that time point.

Both treatments were also associated with an early decline in eGFR (Figure 2A), with a significant reduction from baseline observed with both finerenone (−2.44 mL/min/1.73 m^2^, 95% CI, −4.31 to −0.57) and sMRAs (−2.70 mL/min/1.73 m^2^, 95% CI, −4.77 to −0.63), with no significant between-group difference. However, this reduction was no longer significant at month 12 in the finerenone group, whereas it remained significant in the sMRA group (−4.66 mL/min/1.73 m^2^, 95% CI, −7.35 to −1.97).

Both finerenone and sMRAs were associated with a significant reduction in proteinuria at 12 months (Figure 2B), with percentage changes of −45.63% (95% CI, −59.42 to −27.15) and −44.44% (95% CI, −60.08 to −22.66), respectively, and no significant between-group difference. The reduction was already significant from baseline at 1 month in the sMRA group, whereas in the finerenone group it became significant only at month 6.

SBP (Figure 2C) showed a non-significant decrease in the finerenone group, with a mean change from baseline at month 12 of −3.30 mmHg (95% CI, −10.80 to 4.20). In contrast, sMRAs were associated with a significant reduction in SBP at both 6 months (−5.81 mmHg, 95% CI, −11.45 to −0.17) and 12 months (−10.54 mmHg, 95% CI, −17.77 to −3.32), with a significant between-group difference at month 12 (7.24 mmHg, 95% CI, 2.25–17.66). No significant changes in diastolic blood pressure (DBP) were observed in either group (Figure 2D).

Serum potassium (Figure 2E) increased significantly in both groups at every time point, with no significant between-group difference at month 12 (−0.01 mEq/L, 95% CI, −0.24 to 0.23).

HbA1c (Figure 2F) showed a non-significant increase in the sMRA group and a non-significant decrease in the finerenone group. Nevertheless, at month 12, HbA1c was significantly lower in the finerenone group compared with the sMRA group, with a between-group difference (finerenone minus sMRA) of −7.57 mmol/mol (95% CI, −13.79 to −1.36).

**FIGURE 3:**
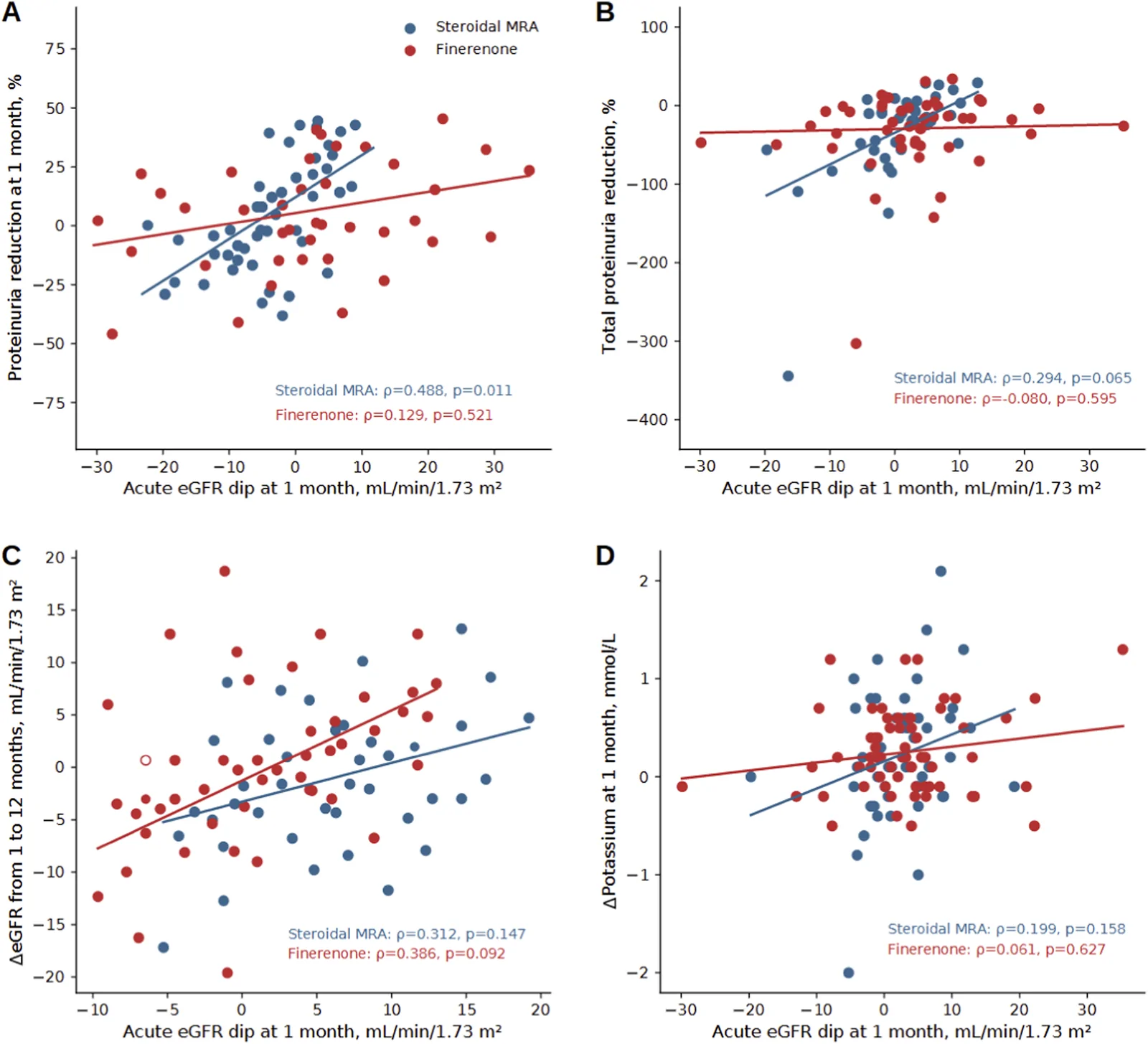
Association between the acute eGFR dip at 1 month and changes in 1-month **(A)** and total **(B)** proteinuria, kidney function **(C)**, and potassium **(D)**.

In correlation analyses (Figure 3), a larger acute eGFR dip from baseline to 1 month was associated with a greater short-term proteinuria reduction in the sMRA group and showed a similar borderline trend for total proteinuria reduction from baseline to the last available follow-up, whereas no association was observed in the finerenone group (1-month proteinuria reduction: sMRA ρ = 0.488, *p* = 0.011; finerenone ρ = 0.129, *p* = 0.521; total proteinuria reduction: sMRA ρ = 0.294, *p* = 0.065; finerenone ρ = −0.080, *p* = 0.595). No significant correlation emerged between the acute eGFR dip and the change in potassium at 1 month in either group (sMRA: ρ = 0.199, *p* = 0.158; finerenone: ρ = 0.061, *p* = 0.627), nor with subsequent eGFR change from month 1 to month 12, although a positive trend was observed, particularly in the finerenone group (sMRA: ρ = 0.312, *p* = 0.147; finerenone: ρ = 0.386, *p* = 0.092).

After adjustment for baseline kidney function, proteinuria, concomitant nephroprotective therapies, and sMRA treatment characteristics, the association between the acute eGFR dip and 1-month proteinuria reduction in the sMRA group was attenuated but remained directionally consistent (partial ρ = 0.416, *p* = 0.068), whereas no association was observed with finerenone (partial ρ = 0.150, *p* = 0.494) (Table 2). Adjustment did not materially alter the absence of associations with total proteinuria reduction, potassium change, or subsequent eGFR change.

**TABLE 2:** Adjusted partial Spearman correlations between acute eGFR dip at 1 month and subsequent clinical changes according to treatment group.

Outcome	Steroidal MRA, partial ρ	P value	Finerenone, partial ρ	P value
Proteinuria reduction, baseline to month 1	0.416	0.068	0.150	0.494
Total proteinuria reduction, baseline to last available follow-up	0.213	0.227	−0.042	0.787
Potassium change, baseline to month 1	0.188	0.253	0.017	0.905
eGFR change, month 1 to month 12	0.050	0.865	0.096	0.743

Partial Spearman correlations were adjusted for baseline eGFR, log-transformed baseline proteinuria, baseline RAAS, inhibitor use, and baseline SGLT2 inhibitor use. In the steroidal MRA, group, analyses were additionally adjusted for MRA, molecule and dose intensity. Finerenone dose was not included because of minimal between-patient variability in dose. Positive values for the acute eGFR, dip indicate a greater decline in eGFR, from baseline to month 1. Total proteinuria reduction was defined as the reduction from baseline to the last available assessment within the 12-month follow-up period.

### 4.4 Exploratory responder analysis

**FIGURE 4:**
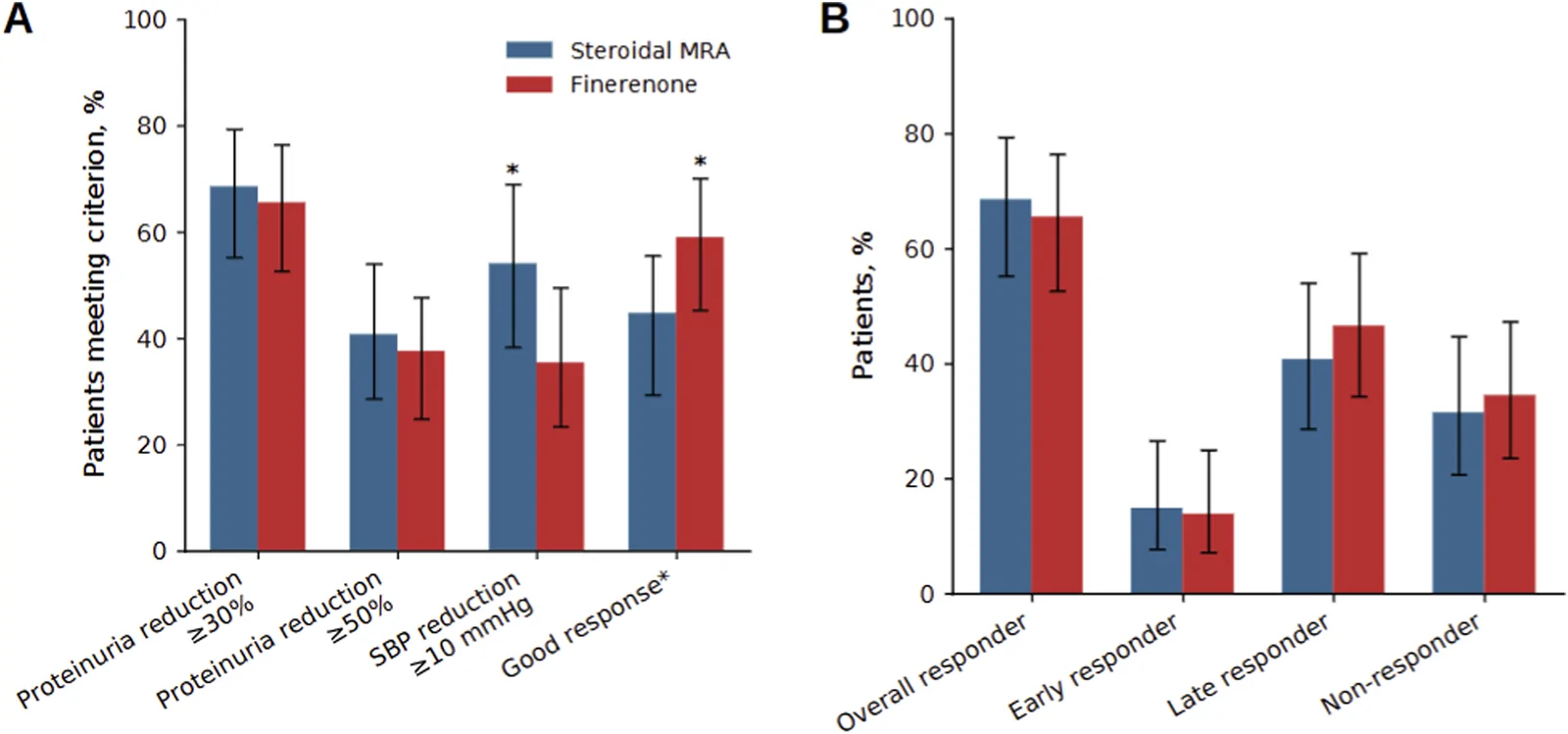
Binary treatment response outcomes and proteinuria responder profile according to treatment group. **(A)** Proportion of patients achieving a ≥30% proteinuria reduction, a ≥50% proteinuria reduction, a ≥10 mmHg SBP reduction, and the composite good renal response. **(B)** Distribution of proteinuria response profiles defined as 30% proteinuria reduction, including overall responders, early responders, late responders, and non-responders.

Both groups showed a comparable antiproteinuric effect (Figure 4), with a similar proportion of patients achieving a ≥30% reduction in proteinuria (65.5% with finerenone and 68.5% with sMRAs). Interestingly, only a small proportion of patients achieved this threshold at month one and were therefore classified as early responders (19.0% with finerenone and 27.8% with sMRAs), whereas most achieved the response later during follow-up and were classified as late responders (46.6% with finerenone and 40.7% with sMRAs). Conversely, 34.5% of patients in the finerenone group and 31.5% in the sMRA group did not achieve a proteinuria reduction of at least 30.0%.

A similar pattern was observed for the achievement of a ≥50.0% reduction in proteinuria, which was reached by a comparable proportion of patients in the two groups (37.5% with finerenone and 40.7% with sMRAs).

For SBP, a ≥10.0 mmHg reduction was achieved more frequently in the sMRA group than in the finerenone group (54.0% vs. 35.4%, respectively), with a significant between-group difference (*p* = 0.034).

For the composite good renal response, defined as a ≥30.0% reduction in proteinuria together with an eGFR decline at 12 months not exceeding 4.0 mL/min/1.73 m^2^, the proportion of responders was significantly higher in the finerenone group than in the sMRA group (59.0% vs. 44.7%, respectively; *p* = 0.046).

## 4.5 Adverse events

**FIGURE 5:**
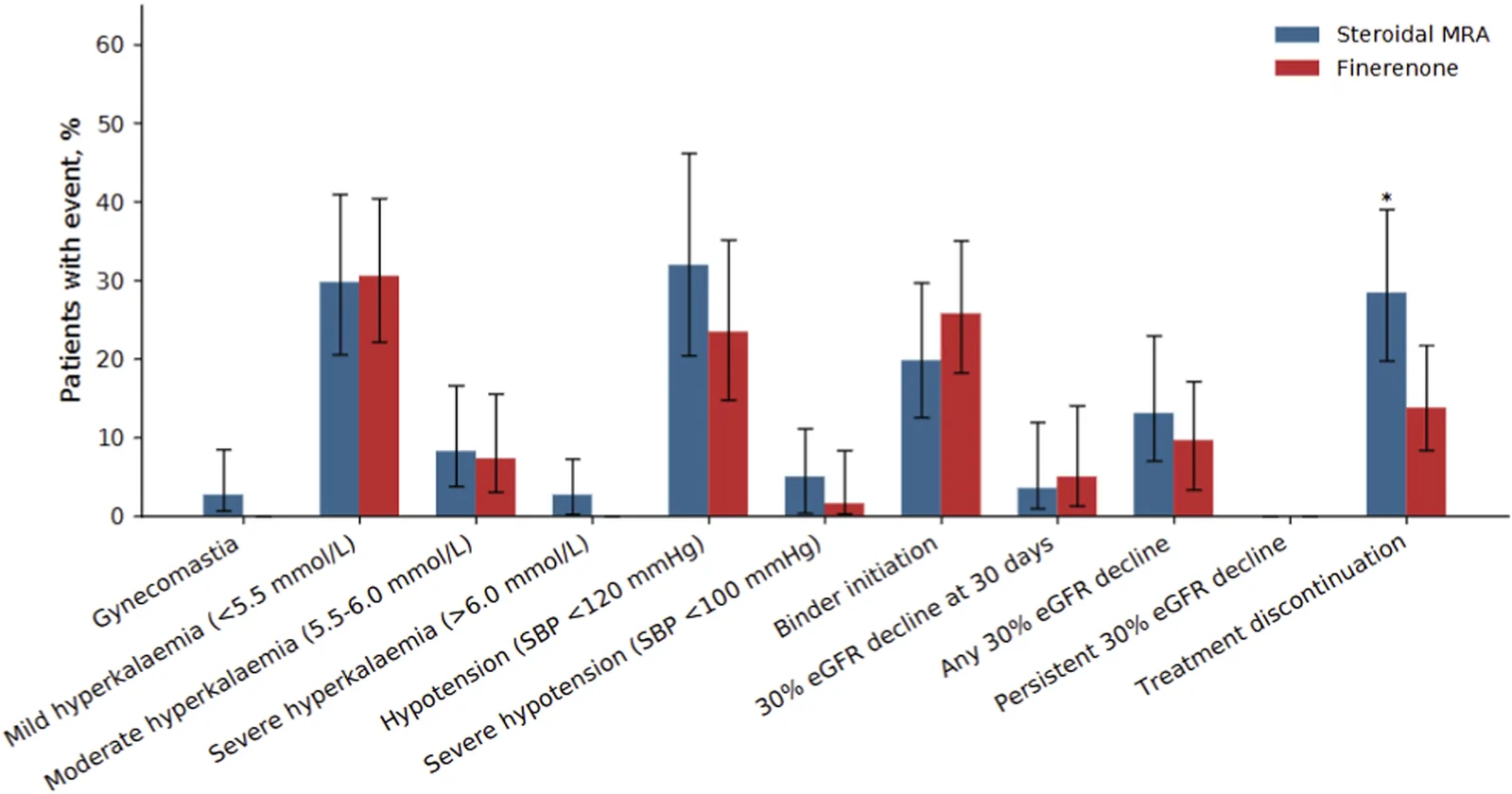
Frequency of adverse events, kidney safety signals, and treatment discontinuation according to treatment group.

Overall, adverse events were broadly comparable between groups, although some differences emerged (Figure 5). Mild and moderate hyperkalemia occurred at similar rates with finerenone and sMRAs (30.7% vs. 29.9% and 7.1% vs. 8.1%, respectively), whereas severe Hyperkalemia was uncommon and observed only in the sMRA group. Gynecomastia was rare and occurred only with sMRAs. Hypotension was less frequent with finerenone than with sMRAs, both for SBP <120 mmHg (23.5% vs. 32.1%) and SBP <100 mmHg (1.2% vs. 4.7%). By contrast, potassium binder initiation was more frequent in the finerenone group (25.8% vs. 19.8%). A 30% eGFR decline at 30 days was infrequent in both groups, although numerically slightly more common with finerenone (5.1% vs. 3.2%). The occurrence of any 30% eGFR decline during follow-up was also relatively uncommon in both groups (9.4% vs. 12.9% with finerenone and sMRAs, respectively), and no patient in either group developed a persistent 30% eGFR decline. Treatment discontinuation was less frequent with finerenone than with sMRAs (13.8% vs. 28.7%, *p* = 0.021).

### 4.5 Sensitivity analyses

In a 1:1 propensity score–matched sensitivity analysis based on age, sex, baseline eGFR, log-transformed baseline proteinuria, diabetes status, and baseline SGLT2 inhibitor use, the main findings remained consistent. After matching, 46 pairs were identified (Supplementary Table S1), and no significant between-group differences were observed in the longitudinal trajectories of either proteinuria or eGFR (group-by-time interaction *p* = 0.802 and *p* = 0.922, respectively) (Supplementary Figure S1). Proteinuria change from baseline at 1 and 12 months was −21.1% (95% CI, −40.3%–4.4%) and −52.2% (95% CI, −68.1% to −28.4%) in the finerenone group, and −30.9% (95% CI, −49.1% to −6.2%), and −47.6% (95% CI, −62.3% to −27.1%) in the sMRA group. Mean eGFR change from baseline at the same timepoints was −2.06 (95% CI, −4.49 to 0.38) and −3.63 (95% CI, −7.07 to −0.20) mL/min/1.73 m^2^ with finerenone, and −2.11 (95% CI, −4.56 to 0.34), and −4.89 (95% CI, −7.85 to −1.93) mL/min/1.73 m^2^ with sMRAs, further supporting the robustness of the primary analysis. The longitudinal patterns of SBP, DBP, and serum potassium were broadly comparable between groups, whereas HbA1c showed a numerically more favorable trend in the finerenone group.

In descriptive sensitivity analyses stratified by individual sMRAs, the overall pattern was broadly consistent with the main findings (Supplementary Table S2; Supplementary Figures S2-S3). In particular, both eplerenone and canrenone showed antiproteinuric activity, but canrenone appeared to be associated with a numerically greater proteinuria-lowering effect than eplerenone, with higher proportions of patients achieving both a ≥30% and a ≥50% reduction in proteinuria. However, this potentially stronger antiproteinuric signal was accompanied by a less favourable safety and hemodynamic profile, as canrenone showed numerically higher rates of hypotension, a greater burden of moderate hyperkalemia, and a more pronounced SBP reduction. Overall, although these subgroup comparisons were descriptive in nature, they largely confirmed the main results while suggesting some heterogeneity within the sMRA group, with canrenone possibly showing a stronger antiproteinuric effect at the expense of lower tolerability.

## 5 Discussion

In this real-world comparative analysis, finerenone and sMRAs showed similar antiproteinuric efficacy, while finerenone appeared to be associated with a more favorable overall profile, including a less pronounced hemodynamic impact, a more favorable eGFR trajectory over follow-up, numerically fewer cases of severe hyperkalemia and gynecomastia, and a lower rate of treatment discontinuation despite more frequent potassium binder initiation.

An additional point worth emphasizing is that, based on the observed dosing patterns, the cohort was exposed predominantly to low-dose MRA regimens over time. Moreover, most patients in the finerenone group had diabetes, which is unsurprising given the current prescribing framework in Italy; accordingly, the few non-diabetic patients receiving finerenone in our cohort were treated off-label. By contrast, although sMRAs are not formally recommended as kidney-protective therapy for proteinuric CKD in current guidelines, in our clinical practice they were widely used in this setting, both in diabetic patients before finerenone became available and in non-diabetic patients, in whom finerenone is still not yet routinely prescribable. This aspect is important when interpreting our comparison, as the sMRA cohort largely reflects an earlier therapeutic era, when finerenone was not yet available and SGLT2 inhibitors were still being progressively incorporated into routine nephrology practice. Similarly, the higher rate of potassium binder initiation in the finerenone group may reflect both the greater availability of these agents in more recent years and a greater tendency to maintain treatment with a guideline-endorsed drug despite manageable hyperkalemia.

The similar antiproteinuric effect observed in our cohort appears clinically meaningful, particularly because patients treated with finerenone had lower baseline proteinuria than those receiving sMRAs. This finding is consistent with randomized evidence showing that finerenone exerts a substantial albuminuria-lowering effect. In FIDELITY (Agarwal et al., 2022), finerenone reduced UACR by 32% versus placebo at 4 months while in CONFIDENCE (Agarwal et al., 2025), finerenone monotherapy showed a clear antiproteinuric effect at day 180 (least-squares mean ratio, 0.68), although the combination with empagliflozin achieved a further reduction. More recently, in FIND-CKD, finerenone also induced a marked antiproteinuric effect, reducing UACR by 41.3% at 6 months, compared with 9.1% with placebo (Heerspink et al., 2026). Conversely, the antiproteinuric efficacy of sMRAs is also well established: in ROTATE-3 (Provenzano et al., 2022), eplerenone reduced UACR by 33.7% after 4 weeks, while broader syntheses reported significant reductions in 24-h proteinuria and UACR with eplerenone and an overall 38.7% reduction in protein/albumin excretion with MR antagonism added to RAS blockade (Hu et al., 2023; Currie et al., 2016). Nevertheless, this observation should be interpreted cautiously in our cohort, as the slight increase in SGLT2 inhibitor use over follow-up in the finerenone group may have introduced some heterogeneity and may have modestly contributed to the observed effect. Conversely, RAAS blockade was already widely used in both cohorts at baseline and remained highly prevalent over time, whereas GLP-1 receptor agonist use was similar between groups and followed a broadly comparable longitudinal pattern.

Interestingly, the correlation analyses suggest that the early eGFR dip may have a different clinical meaning with steroidal and nsMRA. In the sMRA group, a greater acute decline in eGFR was associated with a larger short-term reduction in proteinuria, consistent with a more immediate hemodynamic effect. By contrast, no such relationship was observed with finerenone, whose antiproteinuric effect in our cohort became statistically evident only at month 6, suggesting a slower but more progressive and sustained mechanism of action. Importantly, most nephroprotective therapies—including RAAS inhibitors, SGLT2 inhibitors, and MRA—are associated with an early hemodynamic decline in eGFR, often paralleled by an early reduction in albuminuria and followed by a slower long-term decline in kidney function (Cherney et al., 2026). Nevertheless, although finerenone appears to retain this acute hemodynamic component, its longer-term nephroprotective effect is likely also driven by the broader anti-inflammatory and antifibrotic consequences of selective MR blockade, consistent with the biological profile of nsMRAs. This interpretation is in line with previous evidence indicating that only a minor proportion of finerenone’s clinical benefit is explained by blood pressure lowering alone. Although aldosterone exerts important hemodynamic effects on the kidney, particularly at the level of the efferent arteriole, MR overactivation also promotes vascular remodelling, inflammation, and fibrosis (Uedono et al., 2024; Fioretti et al., 2025). In this context, nsMRAs appear to have a more balanced cardiorenal tissue distribution, whereas sMRAs are thought to exert a more kidney-predominant hemodynamic effect (Agarwal et al., 2021). Supporting this broader biological profile, a *post hoc* biomarker analysis from FIGARO-DKD showed that finerenone modulated circulating proteins involved in RAAS signalling, extracellular matrix remodelling, fibrosis, and neurohormonal pathways, suggesting effects beyond simple hemodynamic changes (Berger et al., 2025).

The blood pressure data also fit well with our findings. In FIDELIO-DKD, finerenone produced only a modest and consistent office SBP reduction, with an overall placebo-adjusted least-squares mean difference of −2.71 mmHg and a maximum difference of −3.84 mmHg at month 4 (Ruilope et al., 2022). Importantly, only 13.8% of the treatment effect on the primary kidney outcome and 12.6% of that on the cardiovascular outcome were explained by office SBP changes, supporting the concept that finerenone’s cardiorenal benefits are only marginally mediated by blood pressure lowering. By contrast, the greater antihypertensive effect observed with sMRAs in our study is pharmacologically plausible. In ASPIRANT-EXT, low-dose spironolactone reduced office SBP by 9.9 mmHg more than placebo at 8 weeks, and 73% of patients achieved an office SBP <140 mmHg versus 41% with placebo (Václavík et al., 2014). Similarly, in the resistant-hypertension subgroup of TOPCAT, spironolactone reduced SBP by 6.1 mmHg and DBP by 2.9 mmHg, with blood pressure control more frequently achieved than with placebo (Rossignol et al., 2018). Even in CKD-specific data, eplerenone lowered SBP by 4.2 mmHg at 4 weeks in ROTATE-3, whereas meta-analytic evidence suggests a more modest average placebo-adjusted SBP reduction with eplerenone overall (Provenzano et al., 2022). All this evidence suggests the need for home blood pressure monitoring after the introduction of this class of drugs into therapy, particularly in normotensive or hypertensive patients on polytherapy for possible adjustments to concomitant therapies.

The potassium findings are likewise coherent with the available literature and probably reflect the main tolerability trade-off within this drug class. In FIDELITY, hyperkalemia-related adverse events were more frequent with finerenone than with placebo (14.0% vs. 6.9%), but permanent discontinuation remained uncommon (1.7% vs. 0.6%), the mean potassium increase after month four was modest (+0.21 mmol/L) (Agarwal et al., 2022). In CONFIDENCE, finerenone monotherapy was associated with potassium >5.5 mmol/L in 18.6% of patients and >6.0 mmol/L in 4.6%, yet adverse events leading to treatment discontinuation remained below 5% (Agarwal et al., 2025). This pattern is consistent with the FINEARTS-HF potassium analysis, in which finerenone increased serum potassium by about 0.19 mmol/L at 1 month and 0.23 mmol/L at 3 months and increased the risk of potassium >5.5 mmol/L, although benefit was maintained under protocol-directed monitoring and dose adjustment (Vardeny et al., 2025). Finallly, in INFINITY, hyperkalemia was more frequent with finerenone than with placebo (14.3% vs. 7.6%), while serious hyperkalemia requiring hospitalisation was uncommon overall (0.9% vs. 0.2%) but appeared to be more pronounced in patients with diabetes than in those without diabetes (Neuen et al., 2026). For sMRAs, ROTATE-3 showed hyperkalemia in 17.4% of patients on eplerenone alone, compared with 4.3% on dapagliflozin-eplerenone combination therapy (Provenzano et al., 2022). Consistently, Hu et al. reported that eplerenone significantly increased serum potassium and the risk of hyperkalemia at both ≥5.5 and ≥6.0 mmol/L thresholds, while Currie et al. found a threefold higher risk of withdrawal due to hyperkalemia with steroidal MR antagonism added to RAS blockade (Hu et al., 2023; Currie et al., 2016). Overall, these data support the interpretation that finerenone retains the class-related potassium signal but with a generally manageable profile, whereas sMRAs appear more hemodynamically active and potentially more difficult to maintain when potassium rises.

Finally, the HbA1c findings deserve a brief comment. We observed a significant between-group difference at month 12, with a decrease in HbA1c in the finerenone group. However, given the retrospective and non-randomized design of the study, this result cannot support any causal or comparative inference, and it would not be appropriate to conclude that finerenone reduced HbA1c more than sMRAs. In addition, we did not have detailed longitudinal data on concomitant glucose-lowering therapies other than GLP-1 receptor agonists; therefore, we cannot exclude the possibility that patients in the finerenone group received a more intensive antidiabetic regimen during follow-up. Nevertheless, this observation is intriguing and may have some biological plausibility. In FINEARTS-HF, finerenone reduced the hazard of new-onset diabetes by 24% compared with placebo in participants without diabetes at baseline, although no meaningful between-group differences in HbA1c were observed over follow-up (Butt et al., 2025). Experimental data also support a potential metabolic effect, as finerenone improved glucose tolerance in a mouse model of high-fat diet-induced obesity by enhancing brown adipose tissue thermogenic activity through activation of the AMPK–ATGL–UCP1 pathway (Marzolla et al., 2020). However, these findings derive from exploratory clinical analyses and preclinical studies; therefore, our HbA1c result should be interpreted as an incidental exploratory observation rather than as evidence of a direct glucose-lowering effect.

Overall, while the observational design precludes causal inference, our findings suggest that finerenone and sMRAs may have different and potentially complementary clinical profiles. Finerenone may be particularly attractive when sustained antiproteinuric efficacy with a limited hemodynamic effect is desirable, whereas sMRAs may offer additional value when greater blood pressure lowering is a therapeutic priority. However, these findings should not be interpreted as supporting any treatment sequence. When MRAs are prescribed primarily for nephroprotection in albuminuric CKD, finerenone remains the preferred evidence-based option, as its cardiorenal benefits have been established in adequately powered randomized outcome trials, whereas comparable kidney outcome trials are lacking for sMRAs. sMRAs may nevertheless represent a reasonable alternative when finerenone is unavailable, unaffordable, not approved or indicated for the individual patient, or otherwise unsuitable. Ultimately, direct head-to-head randomized trials are required to establish the comparative efficacy and safety of steroidal and nonsteroidal MRAs and to inform any definitive treatment hierarchy.

### 5.1 Limitations

This study has several limitations. First, its retrospective, non-randomized design makes it inherently susceptible to selection bias, confounding by indication, and residual confounding. Treatment allocation was driven by clinical practice rather than by protocol, and the two cohorts were not fully comparable at baseline, particularly regarding age, diabetes prevalence, CKD duration, baseline proteinuria, and concomitant therapies. Second, the two treatment groups also reflected, at least in part, different therapeutic eras: the sMRA cohort largely represents an earlier phase of clinical practice, whereas finerenone was introduced later, in a context of broader use of SGLT2 inhibitors, potassium binders, and contemporary nephroprotective strategies. This temporal imbalance may have influenced both efficacy and safety findings. Third, the sample size was limited, especially at later follow-up time points, and the analysis was restricted to patients with available follow-up, thus introducing a potential risk of attrition and survivor bias. Fourth, the sMRA group was itself heterogeneous, including both eplerenone and canrenone, and most patients across groups were exposed to relatively low MRA doses, which may limit the generalizability of the findings. In addition, the use of proteinuria rather than UACR may have slightly reduced comparability with some contemporary studies, which more commonly rely on albuminuria-based definitions and endpoints. However, this choice was necessary within our cohort because, particularly in older clinical records, UACR data were not consistently available, whereas proteinuria measurements were more frequently retrievable. Finally, the finerenone cohort was composed predominantly of patients with diabetes, reflecting current prescribing constraints, which further limits comparability with the sMRA cohort.

These limitations should be carefully considered when interpreting our results. Our analyses were not intended to support inferential or causal conclusions regarding comparative efficacy or safety, but rather to provide a descriptive real-world overview of a cohort exposed to both steroidal and nonsteroidal MRAs in routine nephrology practice. In this context, our findings should be viewed as hypothesis-generating and useful for illustrating patterns of use, longitudinal changes, and tolerability signals in contemporary clinical care.

## 6 Conclusion

In this real-world cohort of patients with proteinuric CKD, finerenone and sMRAs showed broadly comparable antiproteinuric effects over 12 months, supporting the clinical relevance of mineralocorticoid receptor blockade as an add-on strategy in routine nephrology practice. The role of finerenone in CKD is now firmly established by randomized clinical trials demonstrating cardiorenal benefit, particularly in patients with diabetic CKD. Our findings extend this evidence to a real-world setting, showing that finerenone maintained a clinically meaningful antiproteinuric effect while displaying a more favorable tolerability profile than traditional sMRAs, with a less pronounced hemodynamic impact, no observed severe hyperkalemia or endocrine adverse events, and a lower rate of treatment discontinuation.

Importantly, however, finerenone was not devoid of safety concerns. Even in routine clinical practice, MR blockade remained associated with an increased rate of hyperkalemia, which in our cohort was largely managed through initiation of potassium binders rather than treatment withdrawal. These findings highlight that the long-term use of finerenone requires structured biochemical monitoring and proactive potassium management.

Overall, while the observational design precludes causal inference, this study provides a pragmatic snapshot of contemporary clinical practice and suggest that MRA-based therapy, particularly when centered on finerenone, may offer a clinically relevant balance between antiproteinuric efficacy and tolerability in routine nephrology care, emerging as a particularly attractive option when long-term treatment sustainability is a major clinical priority. In this perspective, finerenone may represent not only an effective antiproteinuric option, but also a strategy potentially better aligned with the long-term management needs of patients with CKD.

## Data Availability

The raw data supporting the conclusions of this article will be made available by the authors, without undue reservation.
