# Supplementary material for: A comparative real-world analysis of finerenone and steroidal mineralocorticoid receptor antagonists in patients with chronic kidney disease
Source: Front Pharmacol. 2026 Sep 9;17:1918516. doi: 10.3389/fphar.2026.1918516 (PMC13597784; doi:10.3389/fphar.2026.1918516)
Supplement: Supplementary file 1 [file Supplementaryfile1.docx]

Supplementary Material

**Supplementary Table S1. Baseline characteristics and standardized mean differences in the propensity score–matched cohort.** Caption: Patients were matched 1:1 using propensity score matching based on age, sex, baseline eGFR, log-transformed baseline proteinuria, diabetes status, and baseline SGLT2 inhibitor use. Standardized mean differences (SMDs) are reported to evaluate post-matching covariate balance, with lower values indicating better balance between groups.

| Variable | Steroidal MRA (n=46) | Finerenone (n=46) | SMD |
| --- | --- | --- | --- |
| Matching variables |  |  |  |
| Age | 67.1 ± 12.4 | 67.1 ± 10.8 | 0.004 |
| Female Sex | 7/46 (15.2%) | 11/46 (23.9%) | 0.219 |
| eGFR, mL/min/1.73 m² | 48.9 ± 17.6 | 53.9 ± 24.8 | 0.233 |
| log-Proteinuria | 7.7 ± 0.8 | 7.8 ± 1.0 | 0.084 |
| SGLT2i | 36/46 (78.3%) | 36/46 (78.3%) | 0.000 |
| Diabetes | 42/46 (91.3%) | 42/46 (91.3%) | 0.000 |
| Other variables |  |  |  |
| CKD duration, y | 9.0 [7.0–12.8] | 7.5 [5.0–10.0] | 0.407 |
| SBP, mmHg | 137.5 [120.0–146.2] | 140.0 [130.0–145.0] | 0.112 |
| DBP, mmHg | 80.0 [70.0–80.0] | 80.0 [75.0–80.0] | 0.256 |
| MAP, mmHg | 96.7 [92.9–100.2] | 100.0 [93.3–103.3] | 0.208 |
| Weight, kg | 90.5 [80.0–104.2] | 85.5 [71.2–96.0] | 0.359 |
| BMI, kg/m² | 31.4 [29.5–34.0] | 29.2 [25.9–32.9] | 0.319 |
| Creatinine, mg/dL | 1.5 [1.1–2.0] | 1.6 [1.3–1.8] | 0.130 |
| Proteinuria, mg/24 h | 2660.0 [1666.0–4540.0] | 2087.5 [1500.0–3690.0] | 0.123 |
| Potassium, mmol/L | 4.6 [4.1–4.8] | 4.5 [4.2–4.7] | 0.035 |
| Glucose, mg/dL | 121.5 [98.0–139.2] | 124.0 [98.2–139.8] | 0.043 |
| HbA1c, mmol/mol | 52.5 [48.0–59.0] | 53.0 [48.0–61.0] | 0.188 |
| LDL-C, mg/dL | 71.3 [55.7–104.8] | 66.6 [55.8–97.6] | 0.040 |
| Haemoglobin, g/dL | 13.3 [12.5–14.5] | 14.2 [12.9–15.8] | 0.430 |
| Male sex | 35/46 (76.1%) | 39/46 (84.8%) | 0.219 |
| CVD | 42/46 (91.3%) | 40/46 (87.0%) | 0.140 |
| Hypertension | 45/46 (97.8%) | 45/46 (97.8%) | 0.000 |
| RAASi | 45/46 (97.8%) | 45/46 (97.8%) | 0.000 |
| GLP1-RA | 28/46 (60.9%) | 21/46 (45.7%) | 0.305 |
| DPP4-i | 4/46 (8.7%) | 9/46 (19.6%) | 0.312 |

**Supplementary Table S2. Baseline Characteristics According to Individual Mineralocorticoid Receptor Antagonist Type**

| Variable | Eplerenone  N=46 | Canrenone  N=31 | Finerenone  N=96 | P value |
| --- | --- | --- | --- | --- |
| Age | 69.5 [62.8–73.2] | 68.5 [58.0–73.0] | 72.0 [65.8–77.0] | 0.057 |
| CKD duration, y | 9.5 [7.0–13.0] | 8.0 [5.0–13.0] | 7.0 [4.0–9.5] | 0.01 |
| SBP, mmHg | 135.0 [120.0–150.0] | 130.0 [120.0–145.0] | 130.0 [120.0–140.0] | 0.8 |
| DBP, mmHg | 80.0 [70.0–80.0] | 80.0 [70.0–80.0] | 80.0 [70.0–80.0] | 0.6 |
| MAP, mmHg | 96.7 [91.7–100.0] | 96.7 [90.0–101.2] | 96.7 [90.0–100.0] | 0.9 |
| Weight, kg | 85.0 [75.5–94.4] | 91.0 [76.0–102.0] | 80.0 [69.5–95.9] | 0.2 |
| BMI, kg/m² | 29.7 [24.6–33.2] | 31.4 [30.0–34.4] | 28.7 [25.5–31.4] | 0.072 |
| Creatinine, mg/dL | 1.5 [1.3–2.0] | 1.2 [1.1–1.7] | 1.6 [1.2–1.9] | 0.046 |
| eGFR, mL/min/1.73 m² | 45.7 [29.4–58.9] | 57.2 [40.3–72.0] | 43.0 [34.0–57.8] | 0.03 |
| Proteinuria, mg/24h | 2190.0 [1097.5–3970.0] | 2640.0 [1139.0–4400.0] | 1500.0 [701.2–2607.8] | 0.007 |
| Potassium, mmol/L | 4.5 [4.2–4.8] | 4.5 [3.9–4.8] | 4.6 [4.3–4.9] | 0.6 |
| Glucose*, mg/dL | 119.0 [91.5–143.5] | 117.0 [95.0–130.0] | 125.0 [102.5–140.0] | 0.5 |
| HbA1c*, mmol/mol | 52.5 [46.2–59.0] | 52.0 [48.0–58.0] | 53.0 [48.0–62.5] | 0.6 |
| LDL-C, mg/dL | 67.5 [54.4–104.5] | 88.0 [58.8–124.0] | 65.8 [48.3–83.2] | 0.06 |
| Haemoglobin, g/dL | 13.2 [12.2–14.5] | 13.1 [12.2–14.0] | 13.8 [12.7–15.5] | 0.047 |
| Male sex | 35/46 (76.1%) | 20/31 (64.5%) | 76/96 (79.2%) | 0.2 |
| Diabetes | 29/46 (63.0%) | 22/31 (71.0%) | 89/96 (92.7%) | <0.001 |
| CVD | 35/41 (85.4%) | 22/28 (78.6%) | 81/90 (90.0%) | 0.2 |
| Hypertension | 41/43 (95.3%) | 27/29 (93.1%) | 91/92 (98.9%) | 0.035 |
| RAASi | 40/46 (87.0%) | 25/31 (80.6%) | 87/96 (90.6%) | 0.3 |
| SGLT2i | 29/46 (63.0%) | 17/30 (56.7%) | 66/96 (68.8%) | 0.4 |
| GLP1-RA | 18/46 (39.1%) | 14/31 (45.2%) | 42/96 (43.8%) | 0.8 |
| DPP4-i* | 2/46 (4.3%) | 3/31 (9.7%) | 20/96 (20.8%) | 0.023 |

*Values are expressed as median [interquartile range] or n/N (%), as appropriate. P values refer to comparisons between the steroidal MRA and finerenone groups. Denominators may differ across variables because of missing data. Abbreviations: BMI, body mass index; CKD, chronic kidney disease; CVD, cardiovascular disease; DBP, diastolic blood pressure; DPP4-i, dipeptidyl peptidase-4 inhibitor; eGFR, estimated glomerular filtration rate; GLP1-RA, glucagon-like peptide-1 receptor agonist; HbA1c, glycated haemoglobin; LDL-C, low-density lipoprotein cholesterol; MAP, mean arterial pressure; MRA, mineralocorticoid receptor antagonist; RAASi, renin–angiotensin–aldosterone system inhibitor; SBP, systolic blood pressure; SGLT2i, sodium–glucose cotransporter 2 inhibitor. *available only in patients with Type 2 Diabetes.*

**Supplementary Figure S1. Longitudinal Changes in Clinical and Laboratory Parameters After Propensity Score Matching**

**A.	B.**

**C.	D.**

**E.	F.**

**Supplementary Figure S2. Responder Analysis According to Individual Mineralocorticoid Receptor Antagonist Type**

A.	B.

**Supplementary Figure S3. Safety Outcomes and Treatment Discontinuation According to Individual Mineralocorticoid Receptor Antagonist Type**
